# Mindfulness-Based Cognitive Therapy—Taking it Further (MBCT-TiF) compared to Ongoing Mindfulness Practice (OMP) in the promotion of well-being and mental health: A randomised controlled trial with graduates of MBCT and MBSR^☆^

**DOI:** 10.1016/j.brat.2024.104478

**Published:** 2024-02

**Authors:** Shannon Maloney, Jesus Montero-Marin, Willem Kuyken

**Affiliations:** a Department of Psychiatry, University of Oxford, Oxford, OX37JX, UK; b Teaching, Research & Innovation Unit, Parc Sanitari Sant Joan de Déu, Sant Boi de Llobregat, Spain; c Consortium for Biomedical Research in Epidemiology & Public Health (CIBER Epidemiology and Public Health - CIBERESP), 28029, Madrid, Spain

**Keywords:** Well-being, Mental health, Mindfulness-based, MBCT-TiF, RCT

## Abstract

**Objective:**

To evaluate the effectiveness and acceptability of Mindfulness-Based Cognitive Therapy-Taking it Further (MBCT-TiF), as an adapted programme for graduates of MBCT and Mindfulness-Based Stress Reduction (MBSR). MBCT-TiF sits within a global mental health approach, which aims to help shift a wider distribution of the population towards mental well-being and away from mental ill health using a family of MBCT curricula. The primary hypothesis was that MBCT-TiF, compared to Ongoing Mindfulness Practice (OMP), would help MBCT/MBSR graduates improve their mental well-being.

**Method:**

A parallel RCT with repeated measures was conducted. 164 graduates of MBCT/MBSR were randomly assigned (1:1) to either MBCT-TiF or OMP. Registration: ClinicalTrials.gov (NCT05154266).

**Results:**

Of the 164 graduates recruited, 83 were randomly assigned to MBCT-TiF and 81 to OMP. MBCT-TiF was significantly more effective than OMP at improving mental well-being, with large effects post-intervention (B = 6.25; 95% CI = [4.20, 8.29]; Cohen's *d* = 0.78). No serious adverse effects were reported.

**Conclusions:**

The findings support MBCT-TiF, in the context of the proposed global mental health approach, to help MBCT/MBSR graduates sustain mental health benefits and experience further gains in mental well-being after completing an introductory MBCT/MBSR programme. Future work should consider mechanisms and longer follow-up measurements.

## Highlights

• Results demonstrated effectiveness of MBCT-TiF (vs OMP) in terms of promoting mental health and well-being, for MBCT and MBSR graduates.
• MBCT-TiF (vs OMP) demonstrated reliable improvements in mental well-being with the number-needed-to-treat being 1-in-3 participants.
• MBCT-TiF was deemed acceptable with high ratings of credibility, teacher quality, and engagement.
• Results supported MBCT-TiF in the context of the proposed global mental health approach, called the “pathway for recovery and mental health promotion”.

## 1 Introduction

Mental health conditions continue to be a top leading cause of global disease burden (World Health Organization, 2021). With a higher proportion of total disease burden being attributed to lower-risk cases entering higher-risk categories, without intervention, there is a need for mental health approaches that move beyond treatment and aim to address a wider distribution of the population. Addressing mental health across the population is an important health initiative because it helps individuals and communities build protective factors and greater resilience and protects mental health as fundamental human right for all (Keyes, 2002; Oman, 2023; Patel et al., 2018; United Nations, 2022; World Health Organization, 2022). A series of worldwide challenges (e.g., climate change (Palinkas & Wong, 2020)), economic downturns (Marazziti et al., 2021), and pandemics (Kola et al., 2021) have all adversely affected mental health and there is an urgent need to identify global mental health approaches that can help move the population away from mental ill health and more towards improved mental well-being (Rose, 2008). Given that mental health is shaped by *micro* (i.e., individual, e.g., family history), *meso* (i.e., community, e.g., work or school climate), and *macro* (i.e., global, e.g., poverty) factors, any intervention needs to at minimum consider and ideally target the likely pathways between these factors and mental health.

The field of mindfulness research aligns with global mental health in that both prioritize prevention (in terms of offering “upstream” solutions to reduce the development of mental ill health); aim to strengthen resilience; and recognise the importance of addressing the entire spectrum of mental health, from mental ill health to well-being (Oman, 2023). Recent reviews (Galante et al., 2021; van Agteren et al., 2021) have demonstrated small to moderate effects for mindfulness-based approaches in improving well-being across a wider distribution of the population (e.g., individuals experiencing mental or physical ill health and general population or non-clinical samples). However, despite this promising evidence, the attention to mindfulness-based approaches in the current global health literature is limited (Oman, 2023). Ultimately, more research is required to help build on pre-existing evidence and bridge the conceptual basis for integrating mindfulness-based approaches into global mental health initiatives. Mindfulness-Based Programmes (MBPs) are manualized theory-driven approaches that were introduced in mainstream settings when Mindfulness-Based Stress Reduction (MBSR) was developed to help people with physical health conditions manage symptoms such as chronic pain (Kabat-Zinn, 2013). Past research has demonstrated support for MBSR in improving chronic pain (Cherkin et al., 2016) and symptoms of stress, anxiety, and depression (Fjorback, Arendt, Ørnbøl, Fink, & Walach, 2011). Mindfulness-Based Cognitive Therapy (MBCT), adapted from MBSR, combines psychoeducation elements of cognitive behavioral therapy (CBT) with systematic training in mindfulness meditation (Segal et al., 2018) and was developed specifically to help prevent depression in those at high risk of depressive relapse. Past research has demonstrated effectiveness for MBCT, compared to usual care (e.g., maintenance antidepressants), in reducing depressive relapse (Kuyken et al., 2016). The original MBSR/MBCT protocols aim to target more at-risk population samples and are traditionally formatted as eight-week programmes with weekly group-based sessions (around 1.5 h each), which are led by trained mindfulness teachers, and daily home-based practices (around 30 min per day). However, MBSR/MBCT adaptations have since been developed to help reach wider audiences, using the framework originally proposed by Crane et al. (2017). This framework outlines key elements (‘the warp’; e.g., systematic training in mindfulness), which are retained, and flexible components (‘the weft’; e.g., dosage), which are tailored to unique population samples and contexts to help optimize effectiveness. Some adapted curricula for the general population include MBCT – “Finding Peace in a Frantic World” (M-FP (Williams & Penman, 2011)), MBCT for Life (MBCTL (Strauss et al., 2021)), and MBSR-adapted “Mindfulness-Based College” (MB-College (Loucks et al., 2021)). With the addition of these adapted curricula, MBSR/MBCT have the potential to shift high-risk groups out of the clinical range and into a greater state of wellness with the original MBSR/MBCT protocols (Kabat-Zinn, 2013; Kuyken et al., 2016) and to shift a wider distribution of the population, that currently falls between the cracks of healthcare, towards greater mental well-being.

**Fig. 1:**
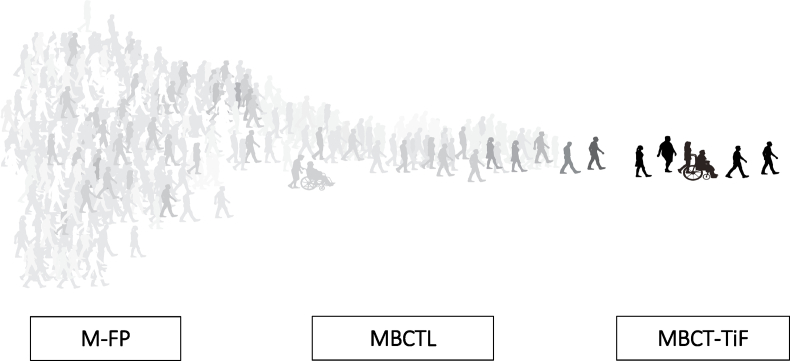
The pathway for recovery and mental health promotion approach Fig. 1. This figure illustrates the pathway for recovery and mental health promotion approach, which outlines a potential care pathway to help shift a wider distribution of the population more towards mental well-being and further away from mental ill health through a family of MBCT curricula adapted for more general population samples. The curricula included within the figure are: Mindfulness-Based Cognitive Therapy-Finding Peace in a Frantic World (M-FP), Mindfulness-Based Cognitive Therapy for Life (MBCTL), and Mindfulness-Based Cognitive Therapy-Taking it Further (MBCT-TiF).

A global mental health approach, that specifically offers a family of MBCT curricula, has been developed whereby a care pathway can be offered to help shift a wider distribution of the population towards mental well-being and away from mental ill health. This global mental health approach, referred to as “the pathway for recovery and mental health promotion”, begins with introductory curricula such as the M-FP programme. M-FP is an accessible low-dose adapted MBCT curriculum, that has reached over two million people worldwide through the dissemination of a book titled: “Mindfulness: A practical guide to finding peace in a frantic world” (Williams & Penman, 2011). This programme, like the original MBCT for Depression protocol, traditionally includes eight weekly group-based sessions, which are led by a trained mindfulness teacher. However, the group-based sessions (e.g., 1 h each) and the home-based mindfulness practices (e.g., 10–15 min per day) are shorter in duration. The idea is that M-FP can be offered at the beginning of the pathway for recovery and mental health promotion (the widest part; Fig. 1) to help reach a wider audience and, therefore, optimize reach. Higher-dose programmes, such as MBCTL, can then be offered which are still framed as introductory but can help deepen understanding. From there, individuals who are looking to find ways of sustaining and extending benefits can advance with the MBCT-Taking it Further (MBCT-TiF) programme. Compared to the original MBCT/MBSR protocols and the adapted MBCT curricula aforementioned (e.g., M-FP and MBCTL), MBCT-TiF traditionally includes twelve weekly group-based sessions (around 1.5–2 h each) and longer home-based mindfulness practices (e.g., 30–45 min per day). In the context of the proposed pathway, the intention is to offer MBCT-TiF to individuals who have already completed an MBCT/MBSR programme (i.e., MBCT/MBSR graduates), positioned at the end of the pathway for recovery and mental health promotion (the narrowest part; Fig. 1), who now have the foundational skills to go deeper with their mindfulness practice. Therefore, while programmes such as M-FP optimize reach, using a low intensity approach, programmes such as MBCT-TiF optimize effects as a result of targeting a population of MBCT/MBSR graduates with a more intensive approach. Overall, the hope is that across this pathway a wider distribution of the population can improve their mental health and well-being. Elements of the proposed pathway have been evaluated; for instance, there are a handful of studies that have examined the effects of M-FP in a range of general population samples (e.g., secondary school teachers, workplace employees) and one study that has evaluated MBCTL in a sample of healthcare workers (de Bruin et al., 2020; Medlicott et al., 2021; Montero-Marin et al., 2021; Strauss et al., 2021). However, this area of research is still in its early stages and no study to date has evaluated the effectiveness and acceptability of MBCT-TiF.

In addition to building on past research, that has evaluated elements of the proposed pathway, there is a need to offer ongoing opportunities that not only help people recover from mental ill health but also help more people realize their full potential in regards to mental well-being. The dissemination of effective mindfulness-based programmes, beyond traditional therapy settings, will be crucial for promoting global mental health. MBCT programmes traditionally follow eight-week formats and, upon completion, individuals are encouraged to continue with their ongoing mindfulness practice to help them sustain mental health benefits over time (Segal et al., 2018; Williams & Penman, 2011). However, qualitative research has indicated that these individuals (i.e., graduates of MBPs) find it challenging to keep up with their practice outside the group context and would welcome further direction on how to extend their learning to better support their mental well-being (Hopkins & Kuyken, 2012). MBCT-TiF was developed with this particular aim in mind, to help graduates of MBCT/MBSR sustain the benefits they acquired in their previous MBP and drive further gains in terms of mental health and well-being. Other MBPs tailored to graduates exist, but they have a particular focus on extending learning in cognate areas (Kramer et al., 2008; Van den Brink & Koster, 2015) or in feeling tone (*vedanā*) (Williams et al., 2022) and are not all explicitly framed within the context of the proposed pathway for recovery and mental health promotion.

The current study sits within a larger area of research that aims to bridge the conceptual basis of integrating MBPs into global mental health initiatives; evaluate the proposed care pathway; and deepen understanding of sustainable ongoing opportunities to better support recovery and mental health promotion. However, the objectives of the current paper are as follows: (1) to provide the first empirical evaluation of an online format of MBCT-TiF in promoting mental well-being [primary outcome], psychological quality of life, and mental health (i.e., symptoms of anxiety and depression) [secondary outcomes]; and (2) to evaluate the acceptability of MBCT-TiF through a wide range of measurements that address expectations, credibility, teacher quality, unpleasant experiences and harm, adverse events, and engagement (attendance, amount of self-led mindfulness practice). The primary hypothesis was that the online format of MBCT-TiF would promote greater mental well-being compared to Ongoing Mindfulness Practice (OMP) in a sample of MBCT/MBSR graduates.

## 2 Method

### 2.1 Study design and participants

We conducted a parallel randomised controlled trial (RCT) with an OMP control arm. Eligibility criteria were assessed by a psychology researcher. Inclusion criteria: Adults aged ≥18 who have completed a formal MBP, which was operationalised as MBCT, MBSR, or any direct adaptation of these parent programmes (‘MBP graduates’), as outlined by Crane et al. (2017). Examples of MBP graduates include individuals who have completed the original MBSR (Kabat-Zinn, 2013) and MBCT for Depression (Segal et al., 2018) protocols or any direct MBCT/MBSR adaptations like M-FP (Williams & Penman, 2011), MBCTL (Strauss et al., 2021), and Mindfulness-Based College (Loucks et al., 2021). Other inclusion criteria included the following: English-speaking and having access to a smart phone or computer to complete online surveys and mindfulness sessions. Exclusion criteria included: Having already completed the MBCT-TiF programme; not completing an MBP that met the aforementioned criteria (Crane et al., 2017); and having already completed teacher training to become a mindfulness teacher.

An MBCT teacher interviewed each participant to ensure they could engage with the MBCT-TiF programme. Participants who indicated recent bereavement, trauma, suicidality, depression, and substance use were contacted by email or phone to discuss the extent to which it was an appropriate time for them to take part. Key factors discussed included: when these experiences took place; whether they had been clinically diagnosed, and if so when; whether the symptoms were ongoing; and what support they had received or were receiving (e.g., medical or clinical intervention, therapeutic or family support). For participants whose symptoms and experiences were current or ongoing, they were excluded from the research study and it was recommended that they take part in the MBCT-TiF programme at a later time and meanwhile seek appropriate professional support.

Participants were recruited and randomised in two study cohorts. Both cohorts were recruited online through email invitation and social media posts, using the Oxford Mindfulness Foundation (OMF) newsletter and social media platforms, and through an existing participant database of MBP graduates who expressed an interest in taking part in future research studies. The first cohort recruited and enrolled individuals in June 2021 (cohort 1) and the second cohort recruited individuals in September 2021 and enrolled individuals in October 2021 (cohort 2). The study was reviewed and approved by the Medical Sciences Interdivisional Research Ethics Committee at the University of Oxford (R75514/RE001; 12/05/2021). All participants provided written informed consent prior to the start of the study. The trial was registered with ClinicalTrials.Gov (NCT05154266; 13/12/2021).

### 2.2 Randomization and masking

Using simple randomization and a computer-generated list, participants were randomly assigned (1:1) to MBCT-TIF or OMP for cohort 1 and cohort 2. A total of 81 participants were recruited in cohort 1 and were randomly assigned to MBCT-TiF or OMP (41 = MBCT-TiF, 40 = OMP), and 83 participants were recruited in cohort 2 and were randomly assigned to MBCT-TiF or OMP (42 = MBCT-TiF, 41 = OMP). Participants in the MBCT-TiF arm were further randomised into groups of equal size (10–14 participants) to standardize group delivery to help control for potential confounding effects.

A member of the study management team, not involved in data collection or analysis, created the randomization list and informed the participants and mindfulness teachers of their allocation by email. Allocation was concealed by the experimenter by having a unique code for each group with the linkage list password-protected and only accessible to the study management team. During the study, concealment of allocation was managed by reminding participants not to email the research team. Moreover, the online surveys only required participants to input their unique code and the linkage list was not shared with the experimenter until after the trial ended. The mindfulness teachers were also asked not to share specific details about a participant during the study unless they had dropped out in light of an adverse event. Data collection was remote and automatic using *Qualtrics* to ensure masking of outcome for the experimenter during the study.

### 2.3 Procedures

#### 2.3.1 Mindfulness-Based Cognitive Therapy–Taking it further

MBCT-TiF is an adapted MBCT curriculum for graduates of MBCT/MBSR and includes the core elements of an MBP (Crane et al., 2017). This programme was developed to help individuals who have already completed an MBP sustain improvements beyond the duration of introductory MBPs and achieve even further gains in terms of mental well-being. In MBCT-TiF, graduates explore weekly themes (i.e., ‘responding not reacting’ and ‘taking care of ourselves and others') which are based on the theoretical map outlined in the book ‘Mindfulness: Ancient wisdom meets modern psychology' (Feldman & Kuyken, 2019). These themes reinforce the learning established in introductory MBCT/MBSR courses whilst also providing new learning opportunities. The programme makes some of the dimensions that are implicit in MBCT/MBSR more explicit; for example, the cultivation of attitudes of mindfulness, such as equanimity, joy, kindness, and friendliness. These new skills require a foundational practice and are thus additive to traditional MBCT/MBSR programmes. MBCT-TiF includes twelve group-based sessions of around 135 min each and daily self-led mindfulness practice of around 30–45 min per day. In the current study, these twelve group-based sessions were held weekly online. These group-based sessions included mindfulness practices, reflections on the self-led home-based mindfulness practice and learning outcomes, and psychoeducation exercises or alternative activities (e.g., poem reading). After each class, participants received an email from their mindfulness teacher about the core learning outcomes and the self-led home-based mindfulness practice.

MBCT-TiF courses were run by certified mindfulness teachers who had taught at least one MBCT-TiF course before the start of the study and who had at least seven years of mindfulness teaching experience. All mindfulness teachers completed MBCT training and met good practice criteria set out by the British Association of Mindfulness-Based Approaches (BAMBA; https://bamba.org.uk/). Peer-led supervision was encouraged on a weekly basis throughout the study. Six different teachers offered the MBCT-TiF course across cohorts. All mindfulness teachers followed the core curriculum by teaching the same mindfulness practices each week and assigning the same self-led home-based mindfulness practice using standardized audio practices. As a form of compensation for taking part in the study, the MBCT-TiF course was significantly subsidized and offered at a 50% discounted rate.

#### 2.3.2 Ongoing mindfulness practice

After completing an MBP, graduates are encouraged to develop an ongoing mindfulness practice whereby they continue their self-led mindfulness practice at home to support their mental health and well-being. While waiting to be offered the MBCT-TiF programme, the OMP control arm continued with their self-led practice during the study period. The amount of self-led practice represented a realistic amount for this sample of MBCT/MBSR graduates. Participants in the OMP arm were encouraged to carry on with the amount of self-led practice that they would normally complete on a daily-basis and were, therefore, not prescribed a specific amount of self-led mindfulness practice.

### 2.4 Outcomes

The primary outcome was mental well-being measured with the 14-item Warwick-Edinburgh Mental Well-being Scale (WEMWBS). The WEMWBS questionnaire covers subjective well-being and psychological functioning. Items are answered on a scale of 1–5 (1 = *None of the time* to 5 = *All of the time*). A total score that ranges 0–40 is interpreted as probable mental health difficulties; 41–44 as possible mental health difficulties; 45–59 as average mental health; and 60–70 as high well-being (https://warwick.ac.uk/fac/sci/med/research/platform/wemwbs/using/howto/). Mental well-being was assessed three times before the start of the twelve-week study period to establish a stable baseline (SB1,SB2) and at pre- and post-intervention. The internal consistency values [Cronbach's alpha (α)] were: SB1: 0.93, SB2: 0.93, Pre: 0.93 and Post: 0.95.

Pre-specified secondary outcomes were considered at pre-post intervention and include psychological quality of life and mental health (i.e., depression and anxiety symptoms). To assess psychological quality of life, the six-item psychological domain of WHOQOL-BREF was used (with items answered on a scale of 1 = *Not at all* to 5 = *An extreme amount*; transformed scores on a 0–100 scale to determine the total score; a total score of 60 and above indicating optimal levels (Silva et al., 2014); and internal consistency values in current study of Pre: 0.83 and Post: 0.87). To assess symptoms of depression, the PHQ-9 was used (with items answered on a scale of 0 = *Not at all* to 3 = *Nearly every day*; symptom severity cut-offs at 5 (*mild*), 10 (*moderate*), 15 (*moderately severe*), and 20 (*severe*); and internal consistency values in current study of Pre: 0.83 and Post: 0.85). To assess symptoms of anxiety, the GAD-7 was used (with items answered on a scale of 0 = *Not at all* to 3 = *Nearly every day*; symptom severity cut-offs at 5 (*mild*), 10 (*moderate*), 15 (*severe*); and internal consistency values in current study of Pre: 0.87 and Post: 0.91).

Measures used to assess acceptability included expectations, credibility, teacher quality, potential unpleasant experiences and harm, and engagement [total attendance and amount of self-led mindfulness practice]. Expectations and credibility were assessed at pre- and post-intervention respectively, with items answered on a scale of 0 = *Not at all* to 10 = *A great deal* and internal consistency values in current study of Pre: 0.88 and Post: 0.89. Overall teacher quality was assessed at post-intervention only (with a single item: ‘Overall, how would you rate the mindfulness teaching you received?’) answered on a scale of 1 = *Incompetent* to 6 = *Outstanding*. Unpleasant experiences and harm were measured at post-intervention only. Engagement (total attendance using teacher-rated attendance sheets and amount of self-led mindfulness practice) was also measured. See Supplement 1 for further details regarding the measures and relevant references and the calculation for the amount of self-led mindfulness practice.

During the study, potential adverse events and harm were monitored and recorded by a trained mindfulness teacher and were reviewed by the Principal Investigator. If the event was deemed related to the intervention, then this event was shared with the ethics committee. For the primary analysis, time points at pre-post intervention were prioritized. Information about process measures (e.g., mindfulness, self-compassion, and decentering) and mid-treatment time points of the primary and secondary outcomes were additionally collected but will be reported elsewhere.

### 2.5 Statistical analysis

The sample size estimation was based on testing whether the trend in pre-post changes differed between groups. The result of the sample size calculation was 168 participants [84 per group] (see Supplement 2 for more details). We described participants’ characteristics at baseline across groups and cohort by means (SD) or frequencies (%), depending on the distribution of each variable.

The primary analysis to assess the effectiveness of MBCT-TiF was developed on an intention-to-treat basis (ITT), with the primary outcome of mental well-being as a continuous variable at the primary endpoint of post-intervention. It involved a repeated measures (RMs) design, using a multilevel mixed effects linear regression model and the restricted maximum likelihood (REML) estimation to account for the correlation between RMs and groups of delivery. Unstandardized slopes and 95% confidence intervals (95% CI) were calculated. Standardized effect sizes were also estimated using Hedges’ *g* from raw data and the combined SD. Effect sizes are considered small when *g* = 0.20, moderate when *g* = 0.50, and large when *g* = 0.80. We used multiple imputations assuming missingness to be at random. Missing outcome data at post-intervention were imputed using chained equations based on linear regressions. The imputation model included the primary and secondary outcomes at previous time points; pre-intervention sociodemographic variables (e.g., age, gender, country, occupation); expectations; type of mindfulness course received before the intervention; amount of self-led mindfulness practice during the intervention; group allocation; and cohort, which generated 20 imputed datasets. Sensitivity analyses were undertaken using complete-cases, and controlling for age, gender, cohort, and previous type of mindfulness course received. The same analytical strategy was used for the secondary outcomes.

Effectiveness was also explored using the Jacobson and Truax method (Jacobson & Truax, 1992) on the primary outcome (i.e., mental well-being) at the primary endpoint (post-intervention). We estimated the standard error (SE) of change and reliable change criterion, calculating reliable improvement, absolute risk reduction (ARR), and number needed to treat (NNT), alongside reliable deterioration as a measure of potential harm effects. The counts (n) and percentages (%) of participants in the well-being categories at post-intervention within the MBCT-TiF and OMP arms were calculated. ARR based on the risk reduction of being in the probable mental health difficulties to average categories (score of 0–59) versus the high mental well-being (score of 60–70) category at post-intervention was also reported along with NNT calculation based on both criteria (i.e., reliable improvement and WEMWBS cut-off for high well-being). The estimated distribution of the raw descriptive means for well-being as a continuous variable from pre-post intervention was plotted within the MBCT-TiF and OMP arms.

Acceptability was explored using means (SD), medians (inter-quartile range (IQR)) or frequencies (%), depending on the distribution of each variable (e.g., expectations, credibility, teacher quality, unpleasant experiences, harm, adverse events, and engagement with the programme [attendance and amount of self-led mindfulness practice]).

### 2.6 Transparency and openness

We have reported our sample size calculation and have outlined data exclusions, where applicable. We have also provided details on the measures we have used following Journal Article Reporting Standard (ARS) (Appelbaum et al., 2018). The data are available upon reasonable request by contacting the corresponding author. Data were analysed using SPSS (v25), *Stata* (v17) and R (v 4.2.1).

## 3 Results

**Fig. 2:**
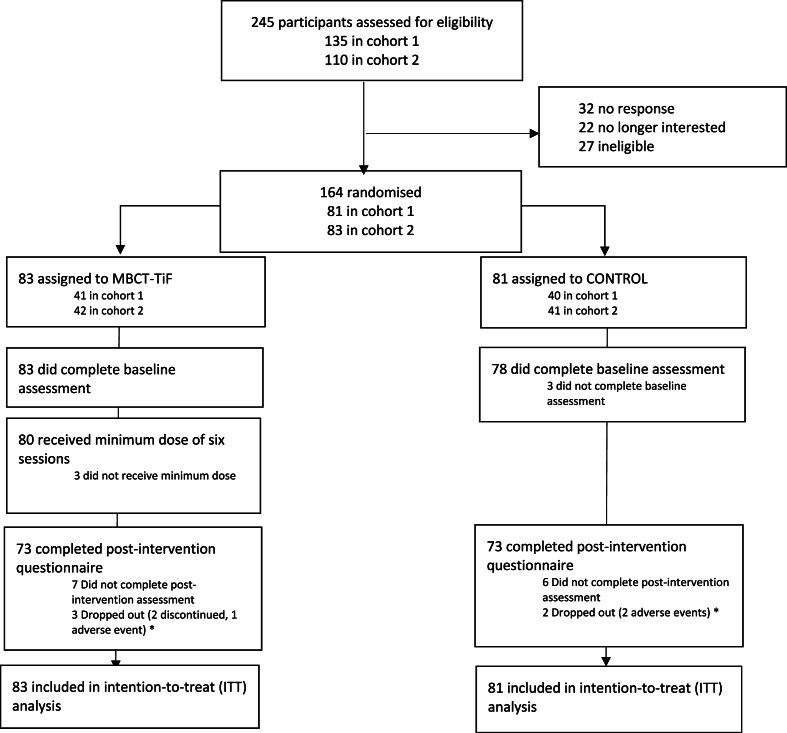
CONSORT Flow Diagram Note. Fig. 2 depicts the CONSORT flow diagram, outlining those assessed for eligibility, randomized, and included for the intention-to-treat (ITT) analysis. The number of those who dropped out, due to discontinuation or adverse event, and who did not complete pre- and/or post-assessments was recorded. Two participants discontinued in the MBCT-TiF arm for the following reasons: no longer eligible and time commitment issue. One adverse event (i.e., hospitalization) was reported in MBCT-TiF arm, which was deemed unrelated to the intervention. Two adverse events were reported in the CONTROL arm (i.e., cancer diagnoses) but were deemed unrelated to the intervention. Cohort 1 was recruited and enrolled in June 2021 and cohort 2 was recruited in September 2021 and enrolled in October 2021. The minimum dose for the MBCT-TiF arm was half (six) of the total (twelve) sessions. MBCT-TiF: Mindfulness-Based Cognitive Therapy–Taking it Further; CONTROL: Ongoing Mindfulness Practice (OMP).

As shown in Fig. 2, of the 164 participants, 83 were randomized to MBCT-TiF and 81 to OMP. Three (3.7%) people in the OMP arm did not complete the baseline measurements and were therefore not included in any analysis. Baseline characteristics were similar between groups (Table 1) and across groups by cohort (Supplement 3). A total of 69.5% identified as female; 74.4% as employed; 67.7% as residing in the United Kingdom; 73.2% as graduates of MBCT; and 26.8% as graduates of MBSR (Table 1). The participants were on average around 51 years old; completed their previous MBP around three years prior to the start of this research study; and reported “average” mental well-being scores (M = 46.72, SD = 8.77). The participants retained at post-intervention had more favorable scores at baseline than those lost to follow-up on mental well-being (47.18 (SD = 8.57) vs 42.94 (SD = 9.69)). These mean scores at baseline were similar between the MBCT-TiF and OMP arms amongst those that followed up post-intervention. Out of those lost at post-intervention (n = 18), cohort 2 reported more attrition (n = 15; 83.3%), compared to cohort 1 (n = 3; 16.7%), which was controlled for in the sensitivity analyses (Supplement 4).

**Table 1:** Baseline characteristics of participants.

	MBCT-TiF (n = 83)	CONTROL (n = 81)	Total (n = 164)
Age, mean (SD)	50.52 (12.50)	50.59 (12.98)	50.55 (12.70)
Gender			
Female, n (%)	56 (67.5)	58 (71.6)	114 (69.5)
Male, n (%)	26 (31.3)	23 (28.4)	49 (29.9)
Country			
UK, n (%)	56 (67.5)	55 (67.9)	111 (67.7)
Others, n (%)	27 (32.5)	26 (32.1)	53 (32.3)
Occupation			
Employed, n (%)	59 (71.1)	63 (77.8)	122 (74.4)
Unemployed, n (%)	4 (4.8)	2 (2.5)	6 (3.7)
Student, n (%)	6 (7.2)	5 (6.2)	11 (6.7)
Retired, n (%)	11 (13.3)	10 (12.3)	21 (12.8)
Previous mindfulness course			
MBCT, n (%)	60 (72.3)	60 (74.1)	120 (73.2)
MBSR, n (%)	23 (27.7)	21 (25.9)	44 (26.8)
Group delivery			
Cohort 1, n (%)	41 (49.4)	40 (49.4)	81 (49.4)
Cohort 2, n (%)	42 (50.6)	41 (50.6)	83 (50.6)
Years since course, mean (SD)	3.65 (3.45)	2.76 (3.44)	3.23 (3.46)
Well-being, mean (SD)	46.58 (9.97)	46.86 (7.40)	46.72 (8.77)

Note. Table 1 includes the baseline characteristics of the ITT sample across groups using means (SD) or frequencies (%), depending on the distribution of the variable. For gender, there was one case in the MBCT-TIF group and no cases in the CONTROL group that identified as ‘other’. For occupation, there were three cases in the MBCT-TIF and one case in the CONTROL group that was missing. For previous mindfulness course (total sample), 44.4% reported the original MBCT for Depression protocol, 25.9% reported MBSR, 23.5% reported MBCTL, 3.7% reported M-FP, and 2.5% reported ‘other’ type of MBCT protocol. A sub-group of the participants completed the question regarding years since completing an MBCT/MBSR course (n = 98). The baseline mental well-being scores with the ITT sample was taken at SB1 time point. MBCT-TiF: Mindfulness-Based Cognitive Therapy – Taking it Further; CONTROL: Ongoing mindfulness practice (OMP).

Of the participants recruited to the trial, 89.0% (88.0% in the MBCT-TiF arm and 90.1% in the OMP arm) provided data on the primary outcome (i.e., mental well-being) at post-intervention. A stable baseline was observed for mental well-being, with no significant between-group differences among the SB1, SB2, and pre-intervention time points (Supplement 5). The result of the between-group analysis according to the mixed effects linear regression model using the ITT sample with imputed data is reported in Table 2. MBCT-TiF was significantly more effective than OMP at improving mental well-being with large effects at post-intervention (B = 5.77; 95% CI = 4.37, 7.17; *d* = 0.78). This result was reinforced both using adjusted means controlling for the covariates, and complete-case analyses (Table 2 and Supplement 6). For the secondary outcomes, MBCT-TiF was significantly more effective than OMP at improving psychological quality of life and symptoms of anxiety and depression with moderate to large effects at post-intervention (Table 2). These results were mirrored both in the adjusted and complete-case analyses (Supplement 6).

**Table 2:** Between-group ITT analyses with imputed data.

Outcomes/Time points	MBCT-TiF M (SD)	CONTROL M (SD)	Hedges' g	B (95% CI)	*p*	Adj-B (95% CI)	*p*
**Primary outcome**							
*Mental well-being*							
Pre-intervention	46.13 (7.73)	46.07 (8.46)					
Post-intervention	52.31 (9.47)	45.91 (9.69)	0.78	5.77 (4.37, 7.17)	<0.001	5.76 (4.36, 7.16)	<0.001
**Secondary outcomes**							
*Quality of life*							
Pre-intervention	55.52 (13.89)	56.55 (14.32)					
Post-intervention	67.61 (16.03)	58.21 (15.86)	0.74	16.90 (14.80, 18.99)	<0.001	16.88 (14.79, 18.98)	<0.001
*Anxiety*							
Pre-intervention	6.61 (3.97)	6.40 (4.26)					
Post-intervention	4.85 (4.02)	6.83 (4.44)	−0.53	−3.21 (−3.77, −2.65)	<0.001	−3.20 (−3.76, −2.64)	<0.001
*Depression*							
Pre-intervention	5.86 (4.50)	6.00 (4.27)					
Post-intervention	4.66 (4.01)	6.74 (4.75)	−0.44	−4.75 (−5.35, −4.14)	<0.001	−4.74 (−5.35, −4.14)	<0.001

Note. Table 2 includes the linear mixed effects regression analyses including groups of delivery and participants as random effects. Descriptive data (M and SD) are raw data, while unstandardized slopes (B) and p-values are adjusted by the linear regression models using imputed data (ITT sample). For the descriptive data, the number of participants were as follows: [MBCT-TiF: Pre = 83, Post = 73; CONTROL: Pre = 78, Post = 73]. For the unstandardized slopes and p-values, using imputed data, the number of participants represent at the point of randomization [MBCT-TiF = 83, CONTROL = 81], with the exception of the three cases from the CONTROL arm that did not complete the baseline measure and were therefore removed from the analysis. The Adj-B values are unstandardized slopes adjusting for the linear regression models and including age, gender, cohort, and previous type of mindfulness course as covariates, using imputed data. Standardized effect sizes were estimated using Hedges' g from raw data by the combined SD weighing the difference in the pre-post means. Mental well-being was measured using 14-item WEMWBS (scores range from 14 to 70; scores 0–40 are interpreted as probable mental health difficulties; 41–44 as possible mental health difficulties; 45–59 as average mental health; and 60–70 as high well-being). Quality of life was measured using the psychological domain of the WHOQOL-BREF (scores range from 0 to 100; scores of 60 and above are interpreted as optimal). Anxiety was measured using the GAD-7 (scores range from 0 to 21 with symptom severity cut-offs at 5 (mild), 10 (moderate), and 15 (severe)). Depression was measured using PHQ-9 (scores range from 0 to 27 with symptom severity cut-offs at 5 (mild), 10 (moderate), 15 (moderately severe), and 20 (severe)). MBCT-TiF: Mindfulness-Based Cognitive Therapy-Taking it Further; CONTROL: Ongoing mindfulness practice (OMP).

Table 3 shows the reliable change for mental well-being (SE of change = 3.28; reliable change criterion = 6.43). A total of 35 participants (47.9%) in the MBCT-TiF arm and 9 participants (12.3%) in the OMP arm experienced a reliable increase in mental well-being between pre-post intervention. The ARR in MBCT-TiF compared to OMP was 35.6% (95% CI = 21.9%, 49.3%), with an NNT = 3 (95% CI = 2.0, 4.6). A total of 4 participants (5.5%) in the MBCT-TiF arm and 11 (15.1%) in the OMP arm experienced a reliable deterioration in mental well-being between pre-post intervention. The NNT to shift into the high mental well-being category at post-intervention was 7 (95% CI = 3.6, 19.4), with an ARR = 16.4% (95% CI = 5.1%, 21.7%) (Supplement 7). The estimated distribution of the raw mental well-being scores pre-post intervention shifted more into the high well-being category (i.e., scores of 60–70) within the MBCT-TiF arm compared to the OMP arm at post-intervention, with 17 (23.0%) shifting into optimal levels within the MBCT-TiF arm and only 5 (6.8%) within the OMP arm (Fig. 2).

**Table 3:** Reliable change on mental well-being.

Reliable Change	RC-		RC0		RC+		TOTAL
Mental well-being	*n*	*%*	*n*	*%*	*n*	*%*	*n*
MBCT-TiF	4	5.5	34	46.6	35	47.9	73
CONTROL	11	15.1	53	72.6	9	12.3	73
TOTAL	15		87		44		

Note*.*Table 3 shows the reliable change analyses for mental well-being. RC-: reliable deterioration. RC0: indeterminate change. RC+: reliable improvement. MBCT-TiF: Mindfulness-Based Cognitive Therapy-Taking it Further. CONTROL: Ongoing mindfulness practice (OMP). Standard error (SE) of change = 3.28; reliable change criterion = 6.43.

Descriptive acceptability ratings for expectations, credibility, teacher quality, unpleasant experiences, and harm within the MBCT-TiF arm are presented in Table 4. Participants in the MBCT-TiF arm reported on average moderately high expectation about the MBCT programme prior to the start of the research study (M = 7.95, SD = 1.44) and reported high levels of credibility after completing the MBCT-TiF programme (M = 8.57, SD = 1.42). On average, the MBCT-TiF arm reported that the teacher quality was “excellent” to “outstanding” (M = 5.18, SD = 0.84). Three participants (1 in MBCT-TiF and 2 in OMP) reported adverse events (2 cancer diagnosis and 1 hospitalization), which were deemed unrelated to the intervention. MBCT-TiF participants attended an average of 10.47 (SD = 2.15) sessions, and a total of 80 participants (96.4% of MBCT-TiF) attended ≥6 sessions. During the study period, the MBCT-TiF arm reported an average of 29.27 (SD = 31.61) minutes of self-led mindfulness practice per day whereas the OMP arm reported an average of 14.43 (SD = 18.87) minutes of self-led home-based practice per day. For more information regarding expectations and frequencies of unpleasant experiences and harm across groups, see Supplement 8.

**Table 4:** Participants’ self-reported acceptability of MBCT-TIF.

Variables	M	SD	Md	IQR
Expectations, range: 0–10 (n = 83)	7.95	1.44	8.20	7.00, 9.20
Credibility, range: 0–10 (n = 73)	8.57	1.42	9.00	7.80, 9.60
Overall teacher quality, range: 1–6 (n = 73)	5.18	0.84	5.00	5.00, 6.00
Unpleasant thoughts/feelings, range: 0–5 (n = 73)	1.16	1.30	1.00	0.00, 2.00
Upsetting experiences, range: 0–3 (n = 73)	0.47	0.58	0.00	0.00, 1.00
Harms, range: 0–3 (n = 73)	0.01	0.12	0.00	0.00, 0.00

Note. Table 4 shows the descriptive data for acceptability ratings for expectations, credibility, overall teacher quality, unpleasant experiences, upsetting experiences, and harm within the MBCT-TiF arm. MBCT-TiF: Mindfulness-Based Cognitive Therapy – Taking it Further; M: mean; SD: standard deviation; Md: median; IQR: interquartile range. Expectations (score range 0–10, ‘not at all’ to ‘a great deal’); Credibility (score range 0–10, ‘not at all’ to ‘a great deal’); Overall teacher quality (score range: 1–6, ‘incompetent’ to ‘outstanding’); Unpleasant thoughts/feelings (score range: 0–5, ‘never’ to ‘daily or almost daily’); Upsetting experiences (score range: 0–3, ‘not at all’ to ‘extremely’); Harm (score range: 0–3, ‘not at all’ to ‘extremely’).

## 4 Discussion

The primary aim of the current study was to evaluate the effectiveness and acceptability of MBCT-TiF, as an adapted programme for MBCT/MBSR graduates. MBCT-TiF sits within a broader global mental health initiative (the pathway for recovery and mental health promotion), which aims to help shift a wider distribution of the population more towards mental well-being and away from mental ill health through a family of MBCT curricula. The current study found that, compared to OMP, the MBCT-TiF programme demonstrated large effects in mental well-being post-intervention. Compared to OMP, one in three individuals that took part in the MBCT-TiF programme experienced reliable improvement in mental well-being (with 1 in every 7 experiencing optimal levels of mental well-being post-intervention). Moreover, the MBCT-TiF programme demonstrated small to moderate improvements in symptoms of anxiety and depression and moderate to large effects for psychological quality of life. Adjusted and complete-case analyses reinforced these findings. Overall, the results demonstrate that MBCT-TiF shifted this sample of MBCT/MBSR graduates further away from mental ill health and more towards mental well-being. Past research, that has examined MBCT programmes that would precede MBCT-TiF (e.g., M-FP) in the context of the proposed pathway for recovery and mental health promotion, has demonstrated similar effects for mental ill health outcomes but smaller effects for mental well-being (Montero-Marin et al., 2021). Therefore, the results of this trial demonstrate potential for MBCT-TiF in providing sustainable mental health benefits and enhancements in mental well-being after completing an MBP. Moreover, this extension of benefits for mental well-being is demonstrated despite comparing MBCT-TiF to an OMP control group. Ultimately, the findings provide support for offering MBCT-TiF to help provide sustainable improvements and even further gains in terms of mental well-being, to follow formats that either address population samples that are either currently at-risk (e.g., original MBCT/MBSR protocols) or a wider distribution of the population that could experience higher levels of well-being (e.g., adapted formats; i.e., M-FP and MBCTL).

MBCT-TiF was also deemed an acceptable programme across a range of ratings (expectations, credibility, teacher quality, unpleasant experiences, harm, and engagement [i.e., attendance and amount of self-led mindfulness practice]). Expectations and credibility ratings were similar to the ratings reported in an instructor-led format of the M-FP programme in a general population sample of UK secondary school teachers (Montero-Marin et al., 2021). Less than 2.0% of the MBCT-TiF arm reported harms, whereas past studies have reported 3.0–7.0% in the context of MBPs and other psychological interventions (Baer et al., 2021; Crawford et al., 2016). Few adverse events were reported in this trial and all were deemed unrelated to the intervention. Across both arms, the attrition rate was less than 4.0% which is extremely low compared to attrition rates in other MBPs (around 17.0%) and CBT (26.2%) (Fernandez et al., 2015; Khoury et al., 2013). This sample was highly engaged in terms of their attendance rates with 96.4% attending at least half the sessions compared to past research which has reported around 86.7% attending at least half the sessions of the M-FP programme (Montero-Marin et al., 2021). However, higher acceptability ratings compared to other mindfulness and psychological interventions are to be expected given that the population of interest concerns MBP graduates who have found MBPs to be beneficial and are therefore more intrinsically motivated to deepen their understanding. Across the pathway for recovery and mental health promotion (Fig. 1), the idea is that introductory curricula (at the beginning of the pathway), like M-FP, can help reach a wider audience and optimize reach whereas more advanced curricula (at the end of the pathway), like MBCT-TiF, can help optimize effects with a targeted sample that now has the foundational skill-set to go deeper with their mindfulness practice.

Global mental health approaches aim to prevent the development of mental ill health and promote improvements in mental-well-being across the population (Rose, 2008). Compared to normative UK data for mental well-being (M = 51.0, SD = 7.0) (Tennant et al., 2007), our sample was within the average range but slightly worse off at baseline (M = 46.72). This could be explained by the fact that 44.4% of our sample had completed MBCT for Depression (MBCT-D) (Segal et al., 2018) prior to the MBCT-TiF programme and, therefore, likely had a history of recurrent depression. Depression is one of the greatest challenges of the 21st century and MBCT, through the original MBCT for depression protocol and this novel MBCT-TiF programme, could potentially help reduce risk of depressive relapse in higher-risk groups but also across the wider population. However, future research will need to consider how to best prevent depression and promote well-being in groups with different risk-status and starting points, in terms of the types of MBPs completed before MBCT-TiF. This work may help determine what works best for whom so that people can be appropriately triaged and effective treatments can be adapted for individual needs. The question regarding differential effects of MBCT-TiF depending on the dosage of the previous MBP (e.g., MFP, MBCTL, MBCTD) will be an important area to explore in future research. Future research will need to investigate this complexity with regard to balancing reach and scalability with impact and the alignment with broader aims associated with a global mental health approach. Overall, the results of this trial sit within a larger body of work that has demonstrated preliminary support for the application of MBCT across a wider distribution of the population (Galante et al., 2021; van Agteren et al., 2021). Past research, that has evaluated mindfulness-based programmes for graduates of MBPs, has reported small to medium effects for mental health outcomes in those at-risk of depressive relapse (Schuling et al., 2020) and a community sample (Williams et al., 2022). However, these programmes differ from MBCT-TiF in terms of the proposed theory of change and/or intention (treatment, prevention, or promotion). Ultimately, MBCT-TiF can be viewed in the context of the proposed pathway for recovery and mental health promotion, which specifically utilizes a family of MBCT curricula, that aims to improve global mental health, whereas other mindfulness-based programmes that target MBP graduates can be viewed as complementary offerings that specifically target the cognate areas (Kramer et al., 2008; Van den Brink & Koster, 2015) or feeling tone (Williams et al., 2022) of mindfulness training.

Several limitations of the present study include: 1) reliance on self-report measures, 2) the wait-list component in the OMP control arm, and 3) lack of follow-up measurement. In the current study, self-report measures were used and future research should consider the addition of more objective measures that also assess functional status. Given that the OMP arm was offered the MBCT-TiF programme at a later time, the wait-list component could have magnified the differences between groups if the control arm had negative expectations as a result of not being offered MBCT-TiF right away. However, the majority of the OMP arm did complete ongoing mindfulness practice during the study period and was therefore not simply waiting to receive the MBCT-TiF programme. The current paper evaluated change pre-post intervention and therefore did not include a follow-up period, which should be included in future work to understand the potential longer-term benefits. Despite these limitations, this trial was methodologically rigorous with the use of randomization and masking of allocation and outcome for the experimenter, and with establishing a stable baseline in the primary outcome. Furthermore, it had excellent retention, it was adequately powered for the primary ITT analyses, and there was very little missing data. To increase external validity, the trial was implemented in a community setting online with trained mindfulness teachers who had extensive teaching experience in this context. The sample was highly selective in that it targets individuals who have benefited from an MBP. However, in the context of the proposed pathway for recovery and mental health promotion, this was the intended population of interest in that the sample is representative of those at the right stage of the proposed pathway to experience further gains in mental health and well-being. Future work can consider collecting additional demographic data (e.g., race, ethnicity, SES, clinical characteristics, current treatment, and treatment history); MBP completion data (e.g., percentage completion of previous MBP or completion degree of previous MBP); and practice data (e.g., frequency of previous mindfulness practice) to further understand effects in different sub-groups of the population that may not have been reached in the current study to help advance our understanding of what works for whom and who may derive benefit from this further training. In light of our research sample targeting MBCT/MBSR graduates who specifically have internet access and availability for synchronous groups sessions, which affects generalizability, future studies should investigate other samples using different formats (e.g., face-to-face versus online). Moreover, future work should consider evaluating mechanisms of action that predict change in mental well-being to help identify components that may be particularly helpful in driving change, which can then be scaled up to help maximize accessibility. Overall, the results of this trial in the context of the wider MBCT literature provide support for the application of MBCT across a wider distribution of the population and in the context of the proposed global mental health initiative (the pathway for recovery and mental health promotion). Depending on where the population sample sits on the spectrum of mental health (e.g., mental ill health to well-being), MBCT can help shift the proportion of the population that is at high-risk of depressive relapse into a greater state of wellness (MBCT for Depression) and a wider distribution of the population that may be relatively well into more optimal ranges of mental well-being through the proposed care pathway. MBCT-TiF can be one key offering that can then help sustain these states of wellness and even drive further gains in mental well-being after completing an MBP. In an effort to address mental health of whole populations, as an important global health initiative in its own right, the results of this trial have provided preliminary support for the proposed pathway that proposes MBCT as one potential offering that can help address the spectrum of mental health and shift a wider distribution of the population more towards mental well-being and away from mental ill health.

## Funding

This research was funded by the 10.13039/100011569Mind & Life Europe Francisco J. Varela Research Award [2020EVA-Maloney, Shannon]. Any views, findings, conclusions, or recommendations expressed in this publication do not necessarily reflect those of Mind & Life Europe. For the purpose of Open Access, the author has applied a CC BY public copyright license to any Author Accepted Manuscript version arising from this submission.

## Acknowlegements

We thank all the mindfulness teachers who took part in the research as well as Alison Yiangou for their input on the course structure and materials. The authors greatly appreciate the help of the Oxford Mindfulness Centre (OMC) administrative and impact teams who helped with scheduling mindfulness courses and liaising with teachers. We thank Alison Burton who helped with randomization and masking and Claire Kelly who spoke with participants to discuss course suitability. The authors also greatly appreciate the funders that have supported the work of individual researchers. For example, Dr. Jesus Montero-Marin has a “Miguel Servet” research contract from the ISCIII (CP21/00080) and is grateful for their support along with the support of CIBER of Epidemiology and Public Health (CIBERESP CB22/02/00052; ISCIII). Professor Willem Kuyken and Dr. Jesus Montero-Marin's work has also been supported by the Wellcome Trust [WT104908/Z/14/Z and WT107496/Z/15/Z]. Most importantly, we thank all the participants who took part in the research trial.

The MBCT-TiF intervention follows the theoretical map outlined in the book titled: ‘*Mindfulness: Ancient wisdom meets modern psychology*’ (Feldman & Kuyken, 2019). Within the methods section, under the description of the MBCT-TiF programme reference to this book is made; however, a formal intervention manual is not yet available.

## CRediT authorship contribution statement

**Shannon Maloney:** Conceptualization, Data curation, Formal analysis, Funding acquisition, Investigation, Methodology, Project administration, Resources, Software, Validation, Visualization, Writing – original draft, Writing – review & editing. **Jesus Montero-Marin:** Conceptualization, Formal analysis, Funding acquisition, Methodology, Validation, Writing – original draft, Writing – review & editing. **Willem Kuyken:** Conceptualization, Formal analysis, Funding acquisition, Methodology, Supervision, Writing – original draft, Writing – review & editing.

## Declaration of competing interest

Professor Willem Kuyken is the Director of the University of Oxford Mindfulness Research Centre. He was until 2015 an unpaid Director of the Mindfulness Network Community Interest Company and since 2014 has been the Director of the Oxford Mindfulness Centre, a collaboration between the University of Oxford and the not-for-profit charity the Oxford Mindfulness Foundation. He receives royalties for several books on mindfulness and CBT published by Guilford Press. Since arriving in Oxford (2014) he has either donated any payments for training workshops and presentations to not-for-profit organisations aligned to his work or used them to fund his research work. Shannon Maloney is a postdoctoral researcher affiliated with the Oxford Mindfulness Centre and does not receive income from this affiliation and therefore declares no financial conflicts of interest. Dr. Jesus Montero-Marin is a senior postdoctoral researcher affiliated with the Oxford Mindfulness Centre but does not receive income from these affiliations and therefore declares no financial conflicts of interest.

## Data Availability

Data will be made available on request.
