# Supplementary material for: Mindfulness-Based Cognitive Therapy—Taking it Further (MBCT-TiF) compared to Ongoing Mindfulness Practice (OMP) in the promotion of well-being and mental health: A randomised controlled trial with graduates of MBCT and MBSR
Source: Behav Res Ther. 2024 Feb;173:104478. doi: 10.1016/j.brat.2024.104478 (PMC11850293; doi:10.1016/j.brat.2024.104478)
Supplement: Multimedia component 1 [file mmc1.docx]

**Mindfulness-Based Cognitive Therapy—Taking it Further (MBCT-TiF) Compared to Ongoing Mindfulness Practice (OMP) in the Promotion of Well-being and Mental Health: A Randomised Controlled Trial with Graduates of MBCT and MBSR**

Shannon Maloney, Jesus Montero-Marin, Willem Kuyken

*Supplementary Information*

**Contents**

**Supplement 1**: Further details on measures and relevant references…………………………..........1

**Supplement 2**: Sample size calculation…………………………………………………….…….….3

**Supplement 3:** Baseline characteristics of participants by cohort………………….………………..4

**Supplement 4**: Selected participant SB1 characteristics by post-intervention status …………….…5

**Supplement 5**: Descriptive statistics and between-group analyses of the primary outcome in the baseline timepoints…………………………………………………...…………………….…….…..6

**Supplement 6**: Complete-case analyses for primary and secondary outcomes…………….…..…....7

**Supplement 7**: Counts and percentages of participants in mental well-being categories at pre-post intervention……..……………………………………………………………………………….....…8

**Supplement 8**: Expectations, unpleasant experiences, and harm across groups………………...…10

**References**……………………………………………………………………………………...…..12

**Supplement 1:** Further details on measures and relevant references

| **Measure** | **Question Examples** | **Reference** |
| --- | --- | --- |
| 14-item Warwick-Edinburgh Mental Well-being (WEMWBS) | “I’ve been feeling optimistic about the future” | Tennant R, Hiller L, Fishwick R, Platt S, Joseph S, Weich S, et al. The Warwick-Edinburgh Mental Well-being Scale (WEMWBS): Development and UK validation. Health Qual Life Outcomes. 2007 Dec; 5(1):63. doi: https://doi.org/10.1186/1477-7525-5-63 |
| World Health Organization Quality of Life-BREF (WHOQOL-BREF) [psychological domain only] | “How much do you enjoy life?” | World Health Organization. Division of Mental Health. WHOQOL-BREF: Introduction, administration, scoring and generic version of assessment: field trial version. 1996 Dec; <https://apps.who.int/iris/handle/10665/63529> |
| Patient Health Questionnaire (PHQ-9) | “Little interest or pleasure in doing things?” | Kroenke K, Spitzer RL, Williams JBW. The PHQ-9: Validity of a brief depression severity measure. J Gen Intern Med. 2001; 16(9):606–13. Doi:  <https://doi.org/10.1046/j.1525-1497.2001.016009606.x> |
| Generalized Anxiety Disorder (GAD-7) | “Feeling nervous, anxious, or on edge?” | Spitzer RL, Kroenke K, Williams JBW, Lowe B. A Brief Measure for Assessing Generalized Anxiety Disorder: The GAD-7. JAMA International Medicine. 2006; 166(10): 1092-1097. doi: 10.1001/archinte.166.10.1092 |
| Expectations and credibility | *Expectations (MBCT-TiF and Control):*   1. “How confident are you that this course will make sense to you?” 2. “How confident are you that this course will affect your well-being?” 3. “How confident would you be in recommending the course to a friend?” 4. “How important do you think it would be to make this course available to others?” 5. “How successful do you believe the course would be in decreasing problems or issues that people have?”   *Credibility (MBCT-TiF only):*   1. “How much did what was taught in the course make sense to you?” 2. “How much do you believe that the course has affected your well-being?” 3. “How confident would you be in recommending the course to a friend?” 4. “How confident would you be in recommending the teacher to a friend?” 5. “How important do you think it would be to make the course available to others?” 6. “How successful do you believe the course was in decreasing problems or issues that people have?” | Montero-Marin J, Taylor L, Crane C, Greenberg MT, Ford TJ, Williams JMG, et al. Teachers “finding peace in a frantic world”: An experimental study of self-taught and instructor-led mindfulness program formats on acceptability, effectiveness, and mechanisms. J Educ Psychol. 2021; 113(8):1689. doi: https://doi.org/10.1037/edu0000542 |
| Unpleasant experiences and harm | *MBCT-TiF:*   1. “How often did the mindfulness course lead to you having unpleasant thoughts or feelings?” on a scale of 0-5 (‘Never’ to ‘Daily or almost daily’) 2. “How upsetting were these experiences?” on a scale of 0-3 ( ‘Not at all’ to ‘Extremely’) 3. “How harmful was the course for you?” on a scale of 1-4 (‘Not at all’ to ‘Extremely’)   *Control:*   1. “How often did your mindfulness practice lead to having unpleasant thoughts or feelings?” on a scale of 0-5 (‘Never’ to ‘Daily or almost daily’) 2. “How upsetting were these experiences?” on a scale of 0-3 ( ‘Not at all’ to ‘Extremely’) 3. “How harmful was the practice you completed over the course of the 12 weeks if at all?” on a scale of 1-4 (‘Not at all’ to ‘Extremely’) | Baer R, Crane C, Montero-Marin J, et al. Frequency of Self-reported Unpleasant Events and Harm in a Mindfulness-Based Program in Two General Population Samples. Mindfulness. 2021 Mar; 12(3):763–74. doi: https://doi.org/10.1007/s12671-020-01547-8 |
| Amount of self-led mindfulness practice per day | Both the MBCT-TiF and OMP arms were asked to report their self-led mindfulness practice during the study period. The average amount of self-led mindfulness practice per day was calculated by multiplying the average number of sitting sessions per week by the average number of minutes per sitting and then divided by seven days. This data was tracked during the study period across both arms and participants were asked to report this data retrospectively on a weekly basis. |  |

Note. Supplement 1 outlines additional question examples and references for measures included in the current paper. For full list of items for 14-item WEMWBS, 6-item psychological domain of the WHOQOL-BREF, PHQ-9, and GAD-7 please review full reference. For expectations and credibility and unpleasant experiences and harm, all items are included in this table. Information regarding how the average amount of self-led mindfulness practice compelted during the study period was calculated is also included.

**Supplement 2:** Sample size calculation

The sample size estimation was based on testing whether the trend in pre-post changes differed between the intervention and control groups. Firstly, we assumed that MBCT-TiF would be able to present moderate effects, compared to the ongoing mindfulness practice (OMP), on the WEMWBS at post-treatment. To operationalize this, we considered a standardized difference between groups on the primary outcome of *g* = 0.48, which corresponds to the average effect observed in a preceding study by our research group with UK secondary teachers self-selected to receive MBCT-L which found WEMWBS means and standard deviations (SDs) at baseline of 49.1 (7.2) (Montero-Marin et al., 2021). The standardized difference also aligns with the usually accepted 0.5 SD criterion which has been considered clinically relevant (Norman et al., 2003). In this preceding study, the inclusion criteria restricted the entry of participants to those interested in mindfulness training, thus we assumed they could be comparable with participants in the present study in the absence of other sources of information on data of graduates from a general population sample. This preceding study and the current study also share a similar active comparator and the samples had similar baseline levels using the 14-item WEMWBS scale. The effect size referred to above corresponds to a difference of 3.6 points in the WEMWBS scale used, which implies an improvement of around 8%. According to the ‘time x group’ interaction in a general linear repeated measures (RM) design, considering the referred difference between the groups, which corresponds to a partial eta square value of roughly 0.06 and an effect size f of 0.25, with a common mean at baseline of 49.1, a mean at post-test of 49.1 and 52.7 respectively, and assuming a correlation of 0.5 across repeated measurements, assuming a common SD of 7.2, a 5% significance level, a statistical power of 80% using a 1:1 ratio, and an univariate approach to RM with Greenhouse-Geisser correction (Abdi, 2010), we needed 64 subjects in each group. Because we expected a dropout rate of approximately 20% (Khoury et al., 2013), and in addition to this, taking a conservative estimate to ensure equal n across the two groups that are of a size that is optimal for learning (i.e., 12 participants per group), we inflated the numbers to reach a total sample size of 168 participants (Green & MacLeod, 2016).

**Supplement 3**: Baseline characteristics of participants by cohort

|  | **Cohort 1**  **(n = 81)** | **Cohort 2**  **(n = 83)** |
| --- | --- | --- |
| Age, mean (SD) | 50.23 (13.1) | 50.87 (12.3) |
| Gender |  |  |
| Female, n (%) | 60 (74.1) | 54 (65.1) |
| Male, n (%) | 21 (25.9) | 28 (33.7) |
| Country |  |  |
| UK, n (%) | 52 (64.2) | 59 (71.1) |
| Others, n (%) | 29 (35.8) | 24 (28.9) |
| Occupation |  |  |
| Employed, n (%) | 60 (74.1) | 63 (75.9) |
| Unemployed, n (%) | 4 (4.9) | 1 (1.2) |
| Student, n (%) | 5 (6.2) | 6 (7.2) |
| Retired, n (%) | 9 (11.1) | 12 (14.5) |
| Mindfulness course |  |  |
| MBCT, n (%) | 62 (76.5) | 58 (69.9) |
| MBSR, n (%) | 19 (23.5) | 25 (30.1) |
| Years since course, mean (SD) | 2.94 (3.8) | 3.57 (3.1) |
| Mental well-being, mean (SD) * | 45.89 (8.8) | 45.78 (8.9) |

Note. Supplement 3 shows the baseline characteristics across groups by cohort status. A sub-sample completed the years since course completion question (52 in Cohort 1 and 46 in Cohort 2). Three cases and one case were missing from occupation question for Cohort 1 and Cohort 2, respectively. One case reported ‘other’ for gender for Cohort 2. Cohort 1 was recruited and enrolled in June 2021 and cohort 2 was recruited in September 2021 and enrolled in October 2021. Three cases were missing from mental well-being scores for Cohort 2.

**Supplement 4**: Selected participant SB1 characteristics by post-intervention status

| **Variables** | **Participants lost at post-intervention** | | | **Remaining participants** | | |
| --- | --- | --- | --- | --- | --- | --- |
|  | **MBCT-TIF**  **(n = 10)** | **CONTROL**  **(n = 8)** | **Total**  **(n = 18)** | **MBCT-TIF**  **(n = 73)** | **CONTROL**  **(n = 73)** | **Total**  **(n = 146)** |
| Age, mean (SD) | 45.10 (11.10) | 52.50 (13.18) | 48.39 (12.29) | 51.26 (12.56) | 50.38 (13.03) | 50.82 (12.76) |
| Gender |  |  |  |  |  |  |
| Female, n (%) | 5 (50.0) | 6 (75.0) | 11 (61.1) | 51 (69.9) | 52 (71.2) | 103 (70.5) |
| Male, n (%) | 5 (50.0) | 2 (25.0) | 7 (38.9) | 21 (28.8) | 21 (28.8) | 42 (28.8) |
| Country |  |  |  |  |  |  |
| UK, n (%) | 5 (50.0) | 6 (75.0) | 11 (61.1) | 51 (69.9) | 49 (67.1) | 100 (68.5) |
| Others, n (%) | 5 (50.0) | 2 (25.0) | 7 (38.9) | 22 (30.1) | 24 (32.9) | 46 (31.5) |
| Occupation |  |  |  |  |  |  |
| Employed, n (%) | 7 (70.0) | 6 (75.0) | 13 (72·.2) | 53 (72.6) | 57 (78.1) | 110 (75.3) |
| Unemployed, n (%) | 0 (0.0) | 0 (0.0) | 0 (0.0) | 3 (4.1) | 2 (2.7) | 5 (3.4) |
| Student, n (%) | 1 (10.0) | 1 (12.5) | 2 (11.1) | 5 (6.8) | 4 (5.5) | 9 (6.2) |
| Retired, n (%) | 2 (20.0) | 0 (0.0) | 2 (11.1) | 9 (12.3) | 10 (13.7) | 19 (13.0) |
| Mindfulness course |  |  |  |  |  |  |
| MBCT, n (%) | 7 (70.0) | 5 (62.5) | 12 (66.7) | 53 (72.6) | 55 (75.3) | 108 (74.0) |
| MBSR, n (%) | 3 (30.0) | 3 (37.5) | 6 (33.3) | 20 (27.4) | 18 (24.7) | 38 (26.0) |
| Group delivery |  |  |  |  |  |  |
| Cohort 1, n (%) | 3 (30.0) | 0 (0.0) | 3 (16.7) | 38 (52.1) | 40 (54.8) | 78 (53.4) |
| Cohort 2, n (%) | 7 (70.0) | 8 (100.0) | 15 (83.3) | 35 (47.9) | 33 (45.2) | 68 (46.6) |
| Years since course, mean (SD) | 1.50 (1.92) | 2.80 (2.78) | 2.22 (2.39) | 3.83 (3.50) | 2.76 (3.54) | 3.34 (3.54) |
| Mental well-being, mean (SD) | 38.70 (9.58) | 48.25 (7.21) | 42.94 (9.69) | 47.66 (9.58) | 46.71 (7.45) | 47.18 (8.57) |

Note. Supplement 4 shows the means (SD) or frequencies of SB1 (baseline) characteristics in those that were lost at T4 and remained in the study at T4. For gender, there was one case in the MBCT-TIF group and no cases in the CONTROL group that identified as ‘other’. For occupation, three cases were missing in the MBCT-TIF group, and no cases were missing in the CONTROL group. For years since course completion, only 89 cases completed this item (48 cases in MBCT-TIF and 41 cases in CONTROL). Only 9 cases (4 in MBCT-TIF and 5 in CONTROL) were reported for years since course completion. MBCT-TiF: Mindfulness-Based Cognitive Therapy; CONTROL: Ongoing mindfulness practice (OMP).

**Supplement 5**: Descriptive statistics and between-group analyses of the primary outcome in the baseline time points

| **Outcome/**  **Time points** | **MBCT-TIF (n = 81)**  M (SD) | **CONTROL (n = 78)**  M (SD) | **Hedges’ g** | **B (95% CI)** | ***p*** |
| --- | --- | --- | --- | --- | --- |
| *Mental well-being* |  |  |  |  |  |
| SB1 | 46.54 (10.01) | 46.81 (7.54) |  |  |  |
| SB2 | 46.57 (9.18) | 46.13 (7.90) | 0.08 | 0.64 (-1.04, 2.33) | 0.454 |
| Pre-intervention | 45.48 (8.80) | 46.24 (8.83) | -0.23 | -0·57 (-2.26, 1.12) | 0.506 |

Note. Supplement 5 includes the linear mixed effects regression analyses including participants as random effects. Descriptive data (M and SD) and effect sizes (*g*) are raw data. Standardized effect sizes were estimated using Hedges’ g from raw data by the combined SD weighing the difference in the pre-post means, while slopes (B) and p-values are adjusted by the linear regression models. MBCT-TIF: Mindfulness-Based Cognitive Therapy-Taking it Further; CONTROL: Ongoing mindfulness practice (OMP).

**Supplement 6**: Complete-case analyses for primary and secondary outcomes

| **Outcome/**  **Time points** | **B (95% CI)** | ***p*** |  | **Adj-B (95% CI)** | ***p*** |
| --- | --- | --- | --- | --- | --- |
| **Primary outcome** |  |  |  |  |  |
| *Well-being* |  |  |  |  |  |
| Pre-post | 6.59 (4.25, 8.93) | < 0.001 |  | 6.58 (4.24, 8.91) | < 0.001 |
| **Secondary outcomes** |  |  |  |  |  |
| *Quality of life* |  |  |  |  |  |
| Pre-post | 11.56 (7.73, 15.38) | < 0.001 |  | 11.53 (7.70, 15.35) | < 0.001 |
| *Anxiety* |  |  |  |  |  |
| Pre-post | -2.43 (-3.62, -1.24) | < 0.001 |  | -2.41 (-3.60, -1.21) | < 0.001 |
| *Depression* |  |  |  |  |  |
| Pre-post | -2.36 (-3.58, -1.13) | < 0.001 |  | -2.34 (-3.57, -1.12) | < 0.001 |

Note. Supplement 6 includes the mixed effects linear regression between-group sensitivity analyses, including participants and groups of delivery as random effects, using a complete case approach (MBCT-TiF: n = 73; CONTROL: n = 73) and controlling for age, gender, previous mindfulness course, and cohort (adjusted models). B: unstandardised regression coefficient (95% CI). Adj-B: unstandardised regression coefficient after adjusting for age, gender, cohort, and previous type of mindfulness course as covariates. Covariates were not significant. MBCT-TiF: Mindfulness-Based Cognitive Therapy -Taking it Further. CONTROL: Ongoing mindfulness practice (OMP).

**Supplement 7**: Counts and percentages of participants on mental well-being categories at pre-post intervention

*Supplementary Table 7a.* Counts and percentages of participants on mental well-being categories at pre-intervnetion

|  | Probable MH difficulties | | Possible MH difficulties | | Average | | High well-being | |
| --- | --- | --- | --- | --- | --- | --- | --- | --- |
|  | *n* | *%* | *n* | *%* | *n* | *%* | *n* | *%* |
| MBCT-TIF (n = 83) | 22 | 26.5 | 10 | 12.0 | 48 | 57.8 | 3 | 3.6 |
| CONTROL (n = 78) | 19 | 24.4 | 15 | 19.2 | 37 | 47.4 | 7 | 9.0 |
| TOTAL (n = 161) | 41 | 25.5 | 25 | 15.5 | 85 | 52.8 | 10 | 6.2 |

Note. Supplementary Table 7a shows the counts and frequencies of mental well-being categories at pre-intervention. The mental well-being categories include those experiencing probable mental health difficulties (score of 0-40), possible mental health difficulties (score of 41-44), average mental health (score of 45-59) and high well-being (score of 60-70). The WEMWBS cut-offs were used. MBCT-TiF: Mindfulness-Based Cognitive Therapy-Taking it Further; CONTROL: Ongoing mindfulness practice (OMP).

*Supplementary Table 7b.* Counts and percentages of participants on mental well-being categories at post-intervention

|  | Probable MH difficulties | | Possible MH difficulties | | Average | | High well-being | |
| --- | --- | --- | --- | --- | --- | --- | --- | --- |
|  | *n* | *%* | *n* | *%* | *n* | *%* | *n* | *%* |
| MBCT-TIF (n = 73) | 8 | 11.0 | 7 | 9.60 | 41 | 56.20 | 17 | 23.30 |
| CONTROL (n = 73) | 23 | 31.50 | 9 | 12.30 | 36 | 49.30 | 5 | 6.80 |
| TOTAL (n = 146) | 31 | 21.20 | 16 | 11.00 | 77 | 52.70 | 22 | 15.10 |

Note. Supplementary Table 7b shows the counts and frequencies of well-being categories at post-intervention. The well-being categories include those experiencing probable mental health difficulties (score of 0-40), possible mental health difficulties (score of 41-44), average mental health (score of 45-59) and high well-being (score of 60-70). The WEMWBS cut-offs were used. ARR was calculated as the percentage risk reduction of the MBCT-TiF group compared to CONTROL in remaining in the probable MH difficulties to average range (score of 0-59) versus high well-being range (score of 60-70) at T4. NNT was calculated using ARR. MBCT-TiF: Mindfulness-Based Cognitive Therapy-Taking it Further; CONTROL: Ongoing mindfulness practice (OMP); ARR: absolute risk reduction; NNT: number needed to treat. ARR = 16.44% (95% CI: 5.1%, 27.7%); NNT = 7 (95% CI: 3.6, 19.4)**.**

**Supplement 8:** Expectations, unpleasant experiences, and harm across groups

*Supplementary Table 8a.* Descriptive statistics and between-group comparison of expectations at pre-intervention

| **Variable/**  **Time point** | **MBCT-TIF (n = 83)**  M (SD) | **CONTROL (n = 78)**  M (SD) | **Effect size**  g | **p-values** |
| --- | --- | --- | --- | --- |
| *Expectations* |  |  |  |  |
| Pre | 7.95 (1.44) | 8.11 (1.46) | 0.11 | 0.483 |

Note. Supplementary Table 8a shows the descriptive data (M and SD) and the between-group comparison using the corresponding t-test for expectations at pre-intervention. Effect sizes were estimated using Hedges’ *g* (small = 0.20, moderate = 0.50, large = 0.80). MBCT-TiF: Mindfulness-Based Cognitive Therapy-Taking it Further; CONTROL: Ongoing mindfulness practice (OMP).

*Supplementary Table 8b.* Counts (%) for unpleasant thoughts/feelings, upsetting experiences and harm across groups

| **Variables at T4** | **MBCT-TiF (n = 73)**  n (%) | **CONTROL (n = 73)**  n (%) | **Total (n = 146)**  n (%) |
| --- | --- | --- | --- |
| **Unpleasant thoughts/feelings** |  |  |  |
| Never | 27 (37.0%) | 23 (31.50%) | 50 (34.20%) |
| Occasionally | 27 (37.0%) | 35 (47.90%) | 62 (42.50%) |
| Less than once/week | 7 (9.60%) | 4 (5.50%) | 11 (7.50%) |
| About once/week | 5 (6.80%) | 4 (5.50%) | 9 (6.20%) |
| Several times/week | 6 (8.20%) | 4 (5.50%) | 10 (6.80%) |
| Daily or almost daily | 1 (1.40%) | 3 (4.10%) | 4 (2.70%) |
| **Upsetting experiences** |  |  |  |
| Not at all | 42 (57.50%) | 44 (60.30%) | 86 (58.90%) |
| Somewhat | 28 (38.40%) | 26 (35.60%) | 54 (37.00%) |
| Quite a bit | 3 (4.10%) | 3 (4.10%) | 6 (4.10%) |
| Extremely | 0 (0.00%) | 0 (0.00%) | 0 (0.00%) |
| **Harms** |  |  |  |
| Not at all | 72 (98.60%) | 72 (98.60%) | 144 (98.60%) |
| Somewhat | 1 (1.40%) | 1 (1.40%) | 2 (1.40%) |
| Quite a bit | 0 (0.00%) | 0 (0.00%) | 0 (0.00%) |
| Extremely | 0 (0.00%) | 0 (0.00%) | 0 (0.00%) |

Note. Supplementary Table 8b shows the count (n) and frequencies (%) for unpleasant thoughts/feelings, upsetting experiences and harm across groups. MBCT-TiF: Mindfulness-Based Cognitive Therapy-Taking it Further; CONTROL: Ongoing mindfulness practice (OMP).

**References**

Abdi, H. (2010). The greenhouse-geisser correction. *Encyclopedia of Research Design*, *1*(1), 544–548.

Green, P., & MacLeod, C. J. (2016). SIMR: An R package for power analysis of generalized linear mixed models by simulation. *Methods in Ecology and Evolution*, *7*(4), 493–498.

Khoury, B., Lecomte, T., Fortin, G., Masse, M., Therien, P., Bouchard, V., Chapleau, M.-A., Paquin, K., & Hofmann, S. G. (2013). Mindfulness-based therapy: A comprehensive meta-analysis. *Clinical Psychology Review*, *33*(6), 763–771. https://doi.org/10.1016/j.cpr.2013.05.005

Montero-Marin, J., Taylor, L., Crane, C., Greenberg, M. T., Ford, T. J., Williams, J. M. G., García-Campayo, J., Sonley, A., Lord, L., Dalgleish, T., Blakemore, S.-J., Team, M., & Kuyken, W. (2021). Teachers “finding peace in a frantic world”: An experimental study of self-taught and instructor-led mindfulness program formats on acceptability, effectiveness, and mechanisms. *Journal of Educational Psychology*, *113*(8), 1689. https://doi.org/10.1037/edu0000542

Norman, G. R., Sloan, J. A., & Wyrwich, K. W. (2003). Interpretation of Changes in Health-Related Quality of Life: The Remarkable Universality of Half a Standard Deviation. *Medical Care*, *41*(5), 582–592.
